# Trajectory Tracking Control for Subsea Mining Vehicles Based on Fuzzy PID Optimised by Genetic Algorithms

**DOI:** 10.3390/s26020441

**Published:** 2026-01-09

**Authors:** Henan Bu, Menglong Wu, Bo Liu, Zhuwen Yan

**Affiliations:** 1 School of Mechanical Engineering, Jiangsu University of Science and Technology, Zhenjiang 212000, China; buhn_just_edu@163.com (H.B.); 15755182470@163.com (M.W.); 18786997619@163.com (B.L.); 2 School of Mechanical Engineering, Nanjing Institute of Technology, Nanjing 211167, China

**Keywords:** deep sea mining vehicle, path following, heading control, fuzzy PID, genetic algorithm

## Abstract

In deep-sea mining operations, the seabed sediments (mud and sand) are very soft and slippery. This often causes tracked vehicles to slip and veer off course when they are driving on the seafloor. To solve the path-tracking problem for deep-sea mining vehicles, this study suggests a path-tracking controller that can adapt to the seabed environment. Firstly, it is necessary to establish a kinematic and dynamic model of the mining vehicle’s motion, analysing its seabed slippage and force application. The system has been developed on the basis of the Stanley algorithm and utilises a two-degree-of-freedom kinematic model, with lateral deviation and heading deviation acting as inputs. The establishment of fuzzy rules to adjust the gain parameter K enables the mining vehicle to adaptively modify its gain parameters according to the seabed environment and path. Secondly, a fuzzy PID controller is established and optimised to address the limitation that fuzzy PID control rules are constrained by the designer’s experience. At the same time, a relationship was established between how fast the drive wheel accelerates and the slip rate based on the dynamic model. This stops the drive wheel from slipping by limiting how fast it can go. Finally, a mechanical model of the mining vehicle was created in Recurdyn and a system model was developed in MATLAB/Simulink for joint simulation analysis. The simulation results demonstrate the efficacy of the proposed control strategy, establishing it as a reliable method for tracking the path of subsea mining vehicles.

## 1. Introduction

As human society continues to develop, land-based mineral resources are being depleted at a rate that is not sustainable in the long term. In contrast, the seafloor contains a wealth of mineable mineral resources, including polymetallic nodules, cobalt-rich crusts and polymetallic sulphides. The seabed mineral resources in question contain a variety of metallic elements, such as copper, nickel and cobalt, which are highly valuable for commercial exploitation [1]. The technology employed in deep-sea mining can be categorised into several key components [2]. These components include: deep-sea mining vehicle technology, hydraulic lift and transport of minerals, mineral collection technology, monitoring and power subsystems, and surface support subsystems. The technology employed in deep-sea mining vehicles constitutes a pivotal element of deep-sea polymetallic nodule mining systems [3]. In the context of mining operations, it is imperative that the mining robot traverses the entire mining area in a comprehensive manner. This is to be achieved through the utilisation of ore collection and ore conveyance apparatus. Nevertheless, the profoundly soft and viscous sediments of the deep seabed render tracked vehicles vulnerable to severe slippage, substantial subsidence, and trajectory deviation. The kinematic performance and control characteristics of these vehicles directly impact their continuous operational performance and operational safety when traversing the seabed.

Currently, research on ground-walking tracked vehicles is relatively mature, with technologies in various aspects well-developed. For ground-walking robots, numerous path tracking methods have been proposed, such as the follow-the-carrot method [4], pure pursuit method [5,6], vector tracking method [7,8], and so on. Although the pure pursuit algorithm is simple and easy to implement, its performance is susceptible to the preview parameter, leading to poor performance under complex working conditions. PID control exhibits excellent robustness in high-speed moving tracked vehicles; however, the parameter tuning process is time-consuming, and it is difficult to obtain optimal parameters [9]. For complex systems such as deep-sea miners, while model predictive control can obtain optimal control parameters in real time and achieve favourable control effects, it involves large computational complexity and is prone to errors caused by model uncertainty, making it challenging to promote and apply in practical engineering projects.

Dai et al. [10] designed a fuzzy PID trajectory tracking controller, constructed a model of a seabed tracked miner using Recursive Dynamic (RecurDyn) software, and applied fuzzy PID for trajectory tracking. The results demonstrated satisfactory performance in straight-line tracking but significant errors during turning transitions. Hong et al. [11] proposed a dynamics-based path tracking control algorithm for underwater tracked vehicles. This algorithm considers track slippage and the longitudinal dynamics model of the vehicle, including the soil-track interaction force model. Bei Xuying et al. [12] designed a suitable sliding mode observer to estimate unknown sliding parameters and reduced the impact of chattering on estimation results through a low-pass filter. Based on Lyapunov’s direct method, they developed the trajectory tracking control law. Jiao Jun et al. [13] designed a sliding mode parameter estimation system based on the Unscented Kalman Filter (UKF), which can provide accurate sliding mode measurements with a high update rate, laying a foundation for the precise control of agricultural tracked robots. Qin et al. [14] proposed a Double-layer Adaptive Unscented Kalman Filter (DAUKF) that can efficiently and accurately estimate the instantaneous centre of rotation of the tracks, thereby enabling the construction of an accurate tracked vehicle model. Compared with other filters, it significantly improves the accuracy of the tracked vehicle model and achieves better trajectory tracking performance. Han et al. [15,16] adopted the PID control algorithm in their pioneering research to realise the goal of seabed tracked mining vehicles travelling along preset paths.

With the in-depth research on the mechanical properties of deep-sea sediment [17,18,19] and the travelling performance of tracked vehicles on soft ground [20,21,22], Dai et al. [23] established a co-simulation model of a seabed tracked vehicle in RecurDyn/Track and MATLAB/Simulink, and proposed an adaptive fuzzy neural control algorithm. Yeu et al. [24] utilised an improved vector tracking path following control algorithm, whose effectiveness was verified through numerical simulations.

Research has indicated that the developed trajectory tracking controller demonstrates optimal performance and effectively mitigates slippage. However, during movement, the presence of varying soil properties can compromise the precision with which traction force and slip ratio can be controlled. This, in turn, may have a detrimental effect on the efficacy of control in operational settings. The present paper investigates the means by which to ensure the adaptability of deep-sea mining vehicles to complex working conditions, with the primary technical contributions outlined as follows: A coupled kinematic and dynamic model of the deep-sea mining vehicle is established, and the dynamic relationship between slip ratio and traction force is derived. A fuzzy gain-adaptive Stanley path-tracking algorithm has been designed, in which a fuzzy logic system has been constructed based on lateral error and heading error to achieve adaptive adjustment of the control gain K, significantly enhancing the adaptability to complex seabed paths. An adaptive genetic algorithm (AGA)-optimised fuzzy PID heading controller has been developed, which globally optimises the fuzzy rule base to balance heading response speed and steady-state accuracy. Concurrently, the angular acceleration of the driving wheels is demonstrably associated with slip characteristics. The system is maintained in the high-adhesion region by the real-time limitation of wheel angular acceleration, thereby ensuring effective suppression of wheel slip. A comprehensive simulation platform has been constructed to validate the effectiveness of the proposed methods under various working conditions, and systematic comparative analyses with conventional control strategies are conducted.

## 2. Deep-Sea Mining Vehicle Model Analysis

In the modelling of deep-sea mining vehicles, commonly employed models include kinematic models and dynamic models. Kinematic models solely investigate the motion characteristics of an object—such as velocity, acceleration, and displacement—without considering the actual forces acting upon it. Dynamic models, however, involve force-driven motion design. Based on Newton’s second law, they consider both the forces acting on an object and its motion in tandem.

The forces acting upon tracked vehicles during motion are highly complex. To simplify the model, the following assumptions are made:

(1) The soil composition remains uniform throughout the mining vehicle’s movement, with identical mechanical properties at all locations. That is, the soil’s mechanical formula remains constant;
(2) Rigid body assumption: The mining vehicle is treated as a single rigid body;
(3) The centres of mass of both tracks and the instantaneous centre of rotation coincide within the xoy plane. That is, during rotation, no lateral slip along the track direction occurs due to the instantaneous centre of rotation not aligning with the track centres of mass;
(4) Errors arising from external disturbances during vehicle motion can be represented by variations in the forces acting upon the vehicle. Consequently, all disturbances can ultimately be synthesised into a single disturbing force acting on the vehicle;
(5) The terrain within the mining vehicle’s operational area exhibits relatively gentle undulations, permitting simplification of its motion to a two-dimensional plane for analysis.

### 2.1. Kinematic Modelling

**Figure 1:**
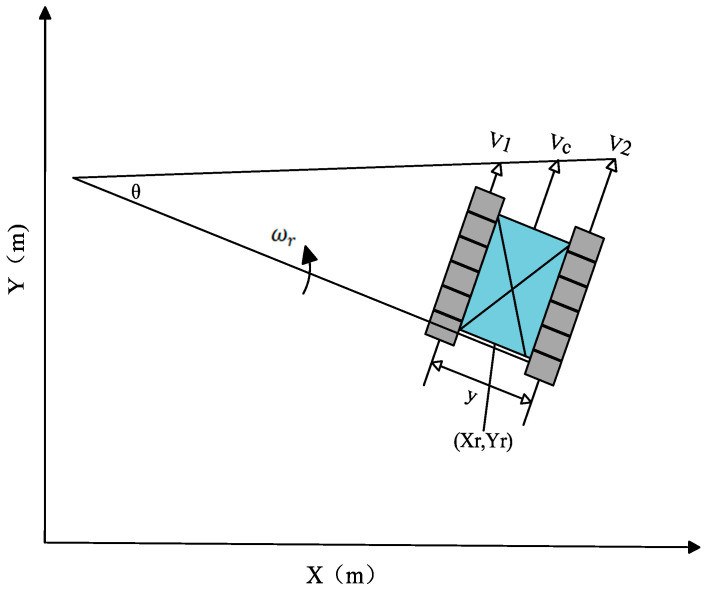
Kinematic model of a mining vehicle.

The deep-sea mining robot adopts a double-track structure, and the kinematic model of a tracked vehicle is usually shown in Figure 1, assuming that the left track speed of the tracked vehicle is $V_{1}$, and the right track speed is $V_{2}$, then the actual speed of the left track centre is designated $V_{1}$, with the direction aligned parallel to the vehicle’s heading, and the actual speed of the right track centre is labelled $V_{2}$, with the direction aligned parallel to the vehicle’s heading. In accordance with the rigid-body assumption, the velocity of the mining vehicle is the average of the velocities of the two sides of the form centre. The steering of the tracked vehicle is achieved by the differential speed of the tracks on both sides.

The kinematic equations are as follows:

(1) $$\left\{\begin{array}{l} V_{c}=\frac{V_{1}+V_{2}}{2}\\ \dot{\theta }=\frac{V_{1}-V_{2}}{Y} \end{array}\right.$$

where $V_{c}$ is the motion speed of the mining robot, m/s; $V_{1}$, $V_{2}$ are the left track speed and right track speed of the mining robot, m/s; $\dot{\theta }$ is the rotational angular speed of the mining robot, rad/s; Y is the centre distance of the track on both sides of the mining robot, m.

Depicted in terms of coordinates travelling, the above equation can be written as:

(2) $$\left\{\begin{array}{l} x(t)=\frac{1}{2}\int_{0}^{t}(V_{1}+V_{2})\cos\theta dt\\ y(t)=\frac{1}{2}\int_{0}^{t}(V_{1}+V_{2})\sin\theta dt\\ \theta =\frac{2}{Y}\int_{0}^{t}(V_{1}-V_{2})\sin\theta dt \end{array}\right.$$

Then the equation of state can be form:

(3) $$\left[\begin{array}{c} \dot{x}\\ \dot{y}\\ \dot{\theta } \end{array}\right]=\left[\begin{array}{c} \frac{1}{2}\sin\theta \frac{1}{2}\cos\theta \\ \frac{1}{2}\sin\theta \frac{1}{2}\sin\theta \\ \frac{2}{Y}-\frac{2}{Y} \end{array}\right]\left[\begin{array}{l} V_{1}\\ V_{2} \end{array}\right]$$

Let x be the state quantity ${\left[x\;y\;\theta \right]}^{T}$, u be the system input ${\left[V_{1}\;V_{2}\right]}^{T}$.

Then the equation of state can be expressed as:

(4) $$\dot{x}=Au$$

where $A=\left[\begin{array}{c} \frac{1}{2}\cos\theta \frac{1}{2}\cos\theta \\ \frac{1}{2}\sin\theta \frac{1}{2}\sin\theta \\ \frac{2}{Y}-\frac{2}{Y} \end{array}\right]$.

### 2.2. Dynamic Modelling

**Figure 2:**
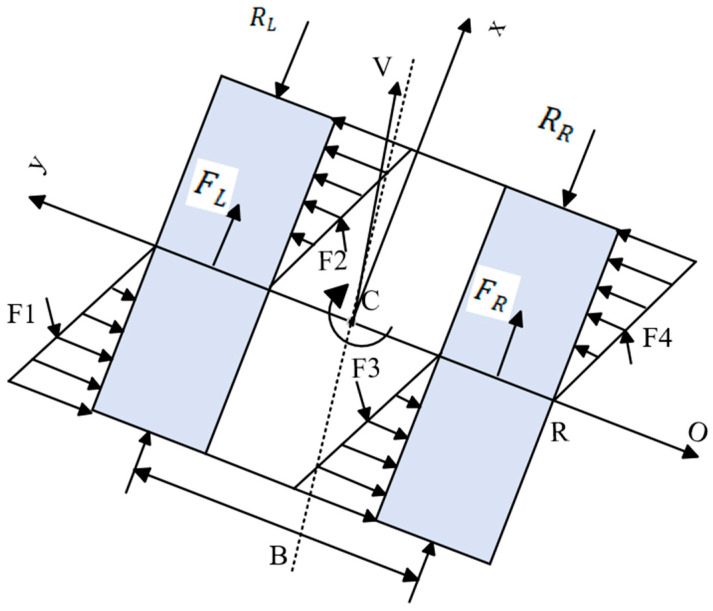
Mining vehicle steering force model.

The dynamics model of the mining vehicle is illustrated in Figure 2, and its dynamics equations are as follows [25]:

(5) $$\left\{\begin{array}{l} M\ddot{x}=F_{L}+F_{R}-R_{L}-R_{R}\\ M\ddot{y}=4F_{y}d\\ I\ddot{\theta }=\frac{(F_{R}-R_{R})D}{2}-\frac{(F_{L}-R_{R})B}{2}-2F_{y}(L^{2}-d^{2}) \end{array}\right.$$

where M is the total mass of the mining robot, kg; $F_{L}$ and $F_{R}$ are the traction force of the left and right tracks, KN, respectively; $R_{L}$ and $R_{R}$ are the resistance forces on the left and right tracks, KN; $F_{1},F_{2},F_{3},F_{4}$ are the lateral resistance forces, KN; Collectively, $F_{y}$, d is the distance between the centre of gravity and the instantaneous centre of rotation, m; B is the track centre distance between the two sides of the mining robot, m; L is the mining robot track grounding length, m.

### 2.3. Analysis of Slip Rate

**Figure 3:**
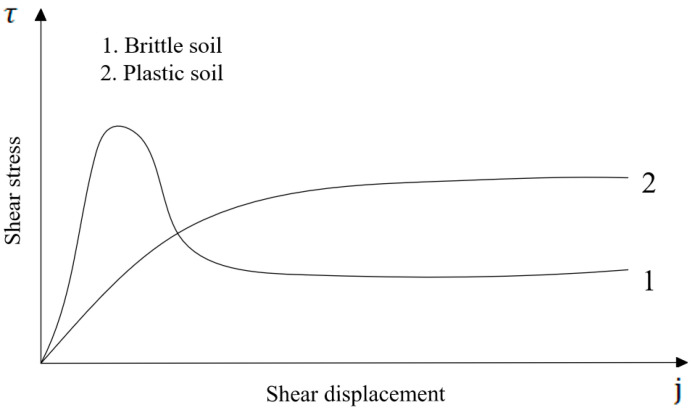
Two types of shear stress-displacement curves.

As demonstrated in Figure 3, there are two prevalent categories of ground shear stress-displacement curves. The first category is characterised by a ground type that extends beyond the hump of the maximum shear stress $\tau_{\max}$ and features a gentle zone of shear stress τ that follows the yield limit. The remaining ground type demonstrates a uniform shear stress-displacement curve, with the shear stress reaching a maximum value and subsequently stabilising.

The study [25] posits that the sea mud in the mining area under consideration is a plastic soil, and that the following equation can be used to calculate the shear stress:

(6) $$\tau =(c+\sigma \tan\varphi )(1-e^{-i/k})=\tau_{\max}(1-e^{-i/k})$$

where c is the cohesion coefficient of the material, Pa; φ is the internal shear strength angle of the material, rad; k is the horizontal shear deformation modulus of the marine mud, m; j is the shear displacement, m; and τ is the shear stress, Pa.

The traction force of a deep-sea mining vehicle is contingent upon the interaction between the vehicle’s tracks and the underlying soil. The process of shearing the soil results in the formation of a track, with the soil itself generating a reverse propulsive force F, which is known as the traction force. The maximum traction $F_{\max}$ depends on the maximum shear strength $\tau_{\max}$ of the ground. Define A as the track grounding area, W as the forward pressure on the vehicle body, σ as the pressure of the vehicle body relative to the ground, and c and φ as the corresponding intra-soil and friction angle parameters. As demonstrated in the study conducted by Bekker M.G. [26], the maximum tensile force that remains in the track is as follows:

(7) $$F_{\max}=A\tau_{\max}=A(c+\sigma \tan\varphi )=Ac+W\tan\varphi$$

It is evident that as the variables of c and φ are subject to variation in accordance with the characteristics of the terrain, the maximum tractive effort experienced by the tracked vehicle is subject to alteration when traversing diverse terrain conditions. In circumstances where the traction required exceeds the maximum traction capacity of the terrain, the tracked vehicle will slip.

Integration of the shear stress along the length of the track has been demonstrated to yield a more accurate formula for the traction force.

(8) $$F=b\int_{0}^{L}\left(c+\sigma (x)\tan\varphi \right)(1-e^{-ix/k})dx$$

where b is the track width, m; l is the track length, m; k is the horizontal shear deformation modulus, m; i is the track slip ratio; and x is the shear displacement, m.

The utilisation of a greater number of smaller support wheels on deep-sea mining vehicles enables the pressure distribution to be approximated as rectangular. Consequently, Formula (8) may be simplified as follows:

(9) $$F=(Ac+W\tan\varphi )\left[1-\frac{k}{il}(1-e^{-il/k})\right]$$

**Figure 4:**
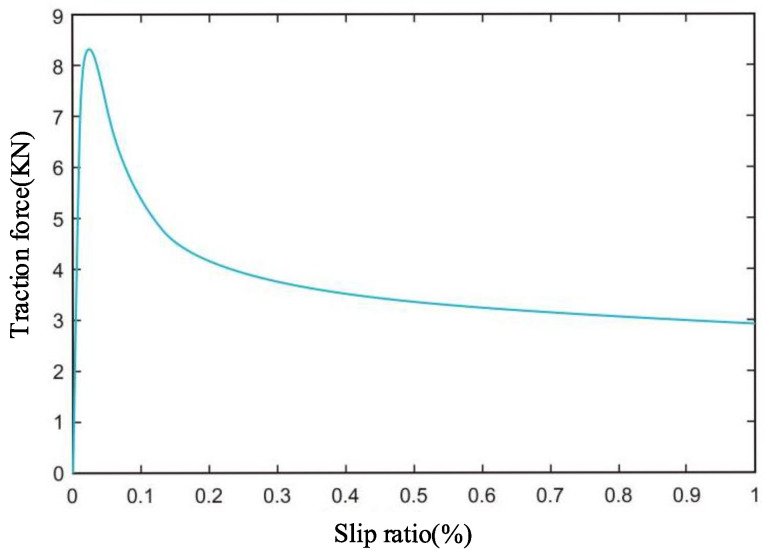
Relationship between mining vehicle traction and slip rate.

The final obtained characteristics of the relationship between track thrust and slippage are shown in Figure 4.

As demonstrated in Figure 4, the soil provides the maximum traction for the mining vehicle at a slip rate of approximately 2.5 per cent, which gradually decreases and stabilises as the slip rate increases. The relationship between soil-provided traction and slip rate was divided into 3 stages based on the variation in soil-provided traction with slip rate: It is evident that during the positive gain phase, the traction force increases in proportion to the increase in slip rate until it reaches its maximum at the process’s peak. This increase in the traction force can be further enhanced by increasing the track speed. In the initial phase, the traction force is observed to decrease in proportion to the increase in slip rate. However, as the slip rate increases, the traction force also increases, reaching a point where it stabilises. Subsequently, an increase in the mining car’s speed results in a decrease in traction force. This, in turn, leads to an increase in slip rate, thereby reversing the control effect. The no-gain phase, in which traction remains essentially constant with increasing slip rate, is a process in which it is not possible to control the mining vehicle by varying the track speed. It can thus be concluded that the most efficacious method of achieving optimal robot motion control is to restrict the mining vehicle’s slippage to within the positive gain phase.

### 2.4. Deep-Sea Mining Vehicle Resistance Model

As the mining vehicle traverses the terrain, it is predominantly subjected to lateral and radial resistance. Lateral resistance can be attributed to the force exerted between the tracks and the ground, while radial resistance is primarily composed of extrusion resistance, water resistance, and thrust resistance. The individual track lateral moments of resistance are:

(10) $$\begin{array}{l} M_{yl}=\int_{0}^{l}xf(x)dx=\int_{0}^{l}x(-\frac{2mg\mu_{l}}{l^{2}}x+\frac{mg\mu_{l}}{l})dx\\ =-\frac{2mg\mu_{l}}{l^{2}}\cdot \frac{l^{2}}{3}+\frac{mg\mu_{l}}{l}\cdot \frac{1}{2}=\frac{mg\mu_{l}}{6} \end{array}$$

where m is the mass of the vehicle body, kg; $\mu_{l}$ is the coefficient of friction; l is the length of the track, m; g is the acceleration of gravity, $\mathrm{kg}/m^{2}$.

It can be demonstrated that, due to the symmetry of the two sides of the track, the total lateral resisting moment $M_{R}$ can be found as follows:

(11) $$M_{R}=2M_{yl}=\frac{mg\mu_{l}}{3}$$

The extrusion resistance is principally generated by the extrusion of the vehicle body weight onto the ground, which exerts a significant influence on the vehicle body’s kinematic performance. Assuming that the normal load is distributed uniformly along the length of the track, the amount of track subsidence can be predicted by the pressure-subsidence equation.

(12) $$R_{1}=\frac{b}{2f}\Delta {\tilde{z}}^{2}-\frac{be}{f}\Delta \tilde{z}$$

where f=1.99−0.112τ
when $\tau \leq 5.0\;\mathrm{kpa},\;e=6.725-2.568\tau +0.245\tau^{2}$
when τ>5.0 kpa, e=0
where b is the width of compacted soil, m; $\Delta \tilde{z}$ is the average value of subsidence, m.

The push resistance of a track of width b against deep-sea land marine mud is:

(13) $$R_{2}=(\frac{1}{2}r_{s}\Delta {\tilde{z}}^{2}k_{pr}+c\Delta {\tilde{z}}^{2}k_{pr})b$$

where $r_{s}$ is the specific gravity of sea mud, $k_{pr}$ and $k_{pc}$ are the passive earth pressure coefficients, which can be calculated according to the following formula:

(14) $$\begin{array}{l} k_{pr}=(\frac{2N_{r}}{\tan\varphi }+1)\cdot \cos^{2}\varphi \\ k_{pc}=(N_{c}-\tan\varphi )\cdot \cos^{2}\varphi \end{array}$$

In the formula, $N_{r}$, $N_{c}$ is the sand-based bearing capacity coefficient. Setting φ to 0.5 allows the ‘too sand-based formula bearing coefficient table’ to be obtained. The resulting values for $N_{r}$ and $N_{c}$ are 0.1 and 6.36, respectively.

Conversely, the resistance of seawater can be estimated by hydrodynamic theory. The hydrodynamic principle posits that the resistance of an object moving in a non-viscous fluid is directly proportional to the surface area of the object, the density of the fluid, and the velocity of the object’s motion. This principle underpins the calculation of resistance in various contexts. So there is:

(15) $$R_{w}=\gamma k_{s}sv/2$$

where γ is the specific gravity of seawater, $k_{s}$ is the scale factor, s is the area of seawater resistance, and v is the vehicle speed.

Therefore, combining the aforementioned equation results in the conversion to the radial resistance of a single track of a mining vehicle, which can be expressed as follows:

(16) $$\begin{array}{l} R=R_{1}+R_{2}+\frac{1}{2}R_{w}\\ =(\frac{b}{2f}\Delta {\tilde{z}}^{2}-\frac{be}{f}\Delta \tilde{z})+(\frac{1}{2}r_{s}\Delta {\tilde{z}}^{2}k_{pr}+c\Delta \tilde{z}k_{pc})b+\frac{\gamma k_{s}sv}{4} \end{array}$$

## 3. Control Algorithm Design

### 3.1. Introduction to Stanley’s Algorithm

The Stanley method is an approach grounded in the compensation of lateral tracking error, which is, in essence, a nonlinear feedback function of the lateral tracking error. The algorithm is characterised by its simplicity and effectiveness, demonstrating robust performance in maintaining stable path tracking under a wide range of road conditions. Additionally, the Stanley method exhibits a high degree of adaptability to different path types, including straight lines, curves and spirals.

The model most frequently employed for path tracking is that of a bicycle, which consists of a steering front wheel, a fixed rear wheel and a connecting rod, with steering performed by the front wheel. In the case of four-wheeled vehicles, the steering is achieved by manipulating the angle of the front wheel. Conversely, in tracked vehicles, the steering is controlled by modulating the rotational speed differential between the tracks on opposing sides.

**Figure 5:**
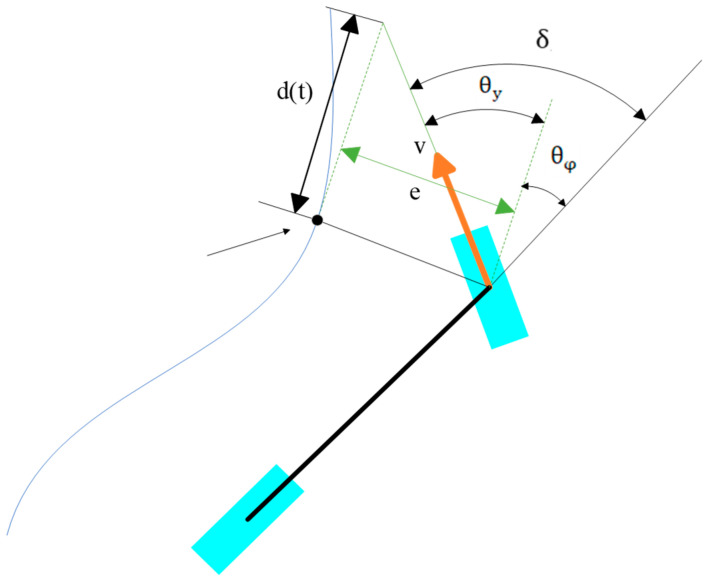
Schematic diagram of Stanley’s method.

As demonstrated in Figure 5, $e_{y}$ denotes the distance from the centre of the front axle to the nearest path point $P_{1}$, i.e., the error value of path tracking. A virtual point is established at the nearest path point $P_{1}$ by extending d(t) along its tangent direction, and the vehicle can be controlled to approach the path by ensuring the front wheels of the vehicle remain pointing in the direction of this virtual point.

### 3.2. Stanley Path Tracking Method for Mining Vehicles

**Figure 6:**
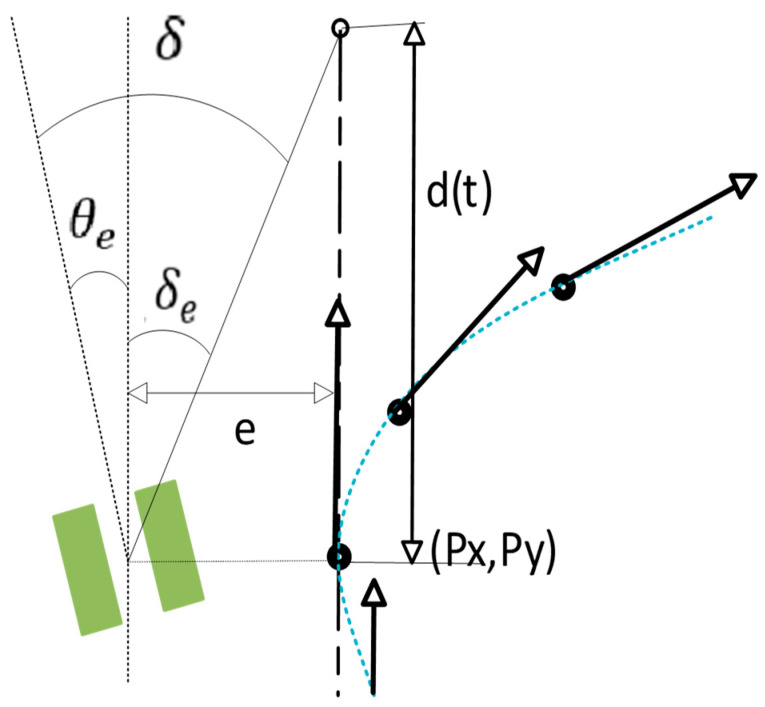
Stanley path tracking method for discrete paths of mining trucks.

The methodology for the trajectory planning of vehicles engaged in deep-sea mining operations, as delineated in this paper, is predicated on a bicycle model. The target point is recalibrated from the closest point to the current position to the subsequent point along the trajectory, in order to adapt to the discrete path. As demonstrated in Figure 6, the virtual point is defined as a point with a distance d(t) from the subsequent point on the trajectory in the direction of the target heading at the next point on the trajectory. In this instance, the direction of e is not necessarily perpendicular to the direction from the virtual target point to the next point on the trajectory, and the principle of the traditional bicycle model cannot be fully applied to the seabed mining vehicle. Consequently, it is imperative to establish a modified version of the Stanley algorithm specifically tailored to the mining vehicle model.

δ is the requisite steering value calculated by the vehicle employing Stanley’s method, $\theta_{e}$ is the discrepancy between the vehicle’s present direction of motion and the tangent direction of the path, and $\delta_{e}$ is the effective steering angle of the vehicle, which satisfy the following relationship equation:

(17) $$\delta =\theta_{e}+\delta_{e}$$

The current position of the mining robot in the geodetic coordinate system is defined as (x,y,θ), the coordinates of the target point as $\left(P_{x},P_{y},P_{\theta }\right)$, e denotes the distance from the mining robot to the target point, and ω denotes the angle between the line connecting the mining robot to the target point and the heading of the target point.

Subsequently, the distance from the mining robot to the virtual target point is as follows:

(18) $$a=\sqrt{e^{2}+d{\left(t\right)}^{2}-2ed(t)}$$

Therefore, the required target heading angle δ of the robot can be expressed as follows:

(19) $$\delta =\theta -\left[P_{\theta }-\arccos\left(\frac{a^{2}+d{\left(t\right)}^{2}-e^{2}}{2ad(t)}\right)\right]$$

### 3.3. Improved Stanley Path Tracking Algorithm

The performance of mining vehicles traversing a designated path is contingent upon their velocity and direction, which is represented by the “gain parameter K”. This is of paramount importance for mining vehicles, as the effectiveness of path-tracking algorithms significantly impacts the vehicle’s overall efficiency. In order to enhance the performance and stability of path-tracking systems, this paper proposes a novel combination of the robust and adaptive properties of fuzzy logic with the traditional Stanley controller. The employment of fuzzy logic to optimise the gain coefficient K of the Stanley path-tracking algorithm results in the derivation of an adaptive Stanley path-tracking algorithm, which serves to enhance the accuracy of tracking.

#### 3.3.1. Fuzzification

The design of a control system that can adaptively adjust the gain parameter K is presented, with inputs taken from the lateral deviation and heading deviation of the mining vehicle, and the output being the gain parameter K. The specific process is as follows: the basic domain of lateral deviation e: {−2.5 m, 2.5 m}; the quantification level: {−6, −5, −4, −3, −2, −1, 0, 1, 2, 3, 4, 5, 6}; the basic domain of heading deviation $\theta_{e}$: {−60°, 60°]; the quantification level: {−6, −5, −4, −3, −2, −1, 0, 1, 2, 3, 4, 5, 6}; the gain parameter K is the input to the mining truck. The fundamental domain of the gain parameter K is defined as [0, 10], with the quantisation level set to {−6, −5, −4, −3, −2, −1, 0, 1, 2, 3, 4, 5, 6}.

It is demonstrated that seven linguistic variables can express the fuzzy subsets of heading deviation, lateral deviation and gain parameter with sufficient accuracy. The fuzzy levels of heading deviation and lateral deviation are negative big (NB), negative medium (NM), negative small (NS), zero (ZO), positive small (PS), positive medium (PM) and positive big (PB); and the fuzzy levels of the gain parameter K are very big (VB), big (B), big (CD), moderate (M), small (CS), small (S) and very small (VS). In this case, the implementation of Gaussian functions is employed for the modelling of heading and lateral errors, whilst trigonometric functions are utilised for the modelling of gain parameters.

#### 3.3.2. Fuzzy Rulemaking

**Figure 7:**
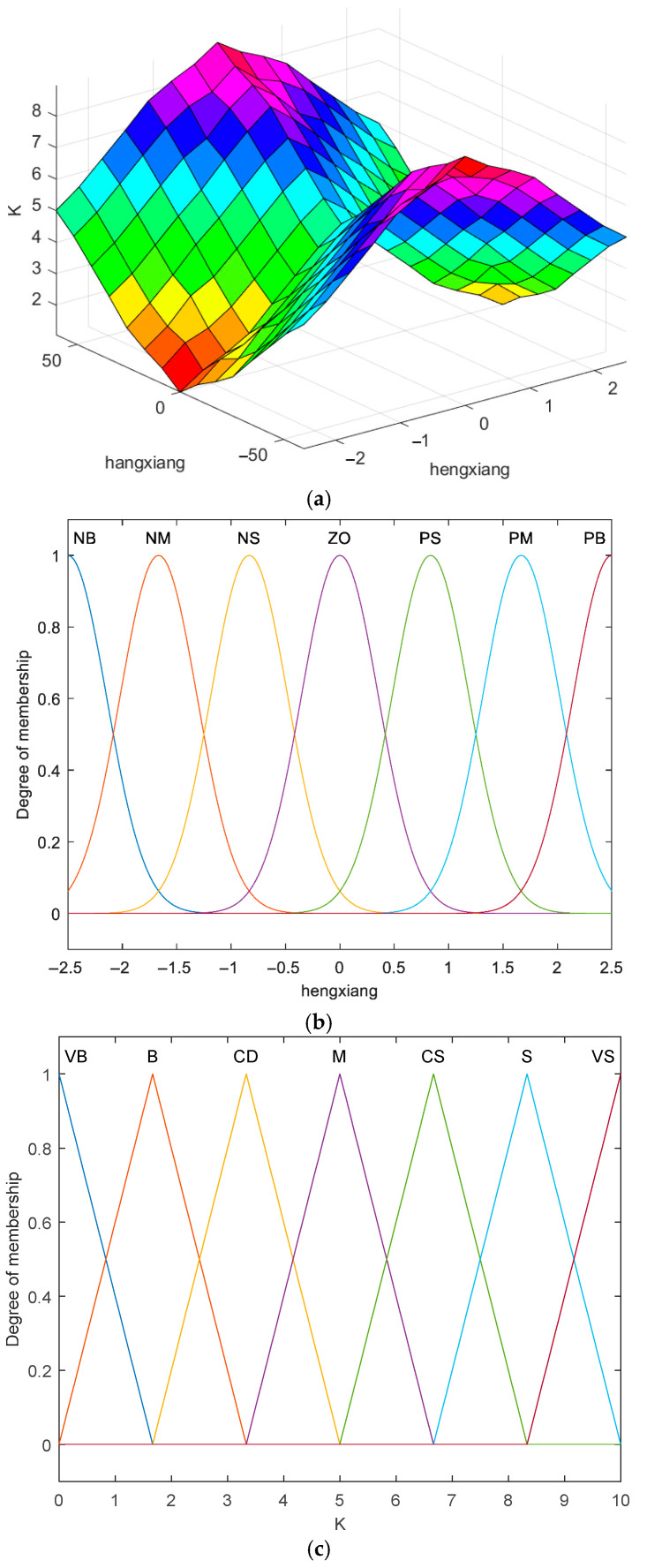
(**a**) Gain parameter K; (**b**) Enter the affiliation function; (**c**) Output affiliation function.

Fuzzy rules constitute the fundamental components of a fuzzy logic system, which emulates the imprecision characteristic of human daily language. These systems are employed to address uncertainty and ambiguity in information processing. The formulation of fuzzy inference rules is predicated on the correspondence of each variable. The following set of fuzzy rules, formulated through trial and error and alternative methods, elucidate the effect of heading deviation on the magnitude of gain parameters:

(1) In accordance with the established practices concerning land vehicle travel, it is imperative that the gain parameter *K* is not unduly large when the lateral deviation is substantial, as this may precipitate acute turns and consequently result in accidents. Conversely, when the lateral deviation is minimal, it is essential to augment the gain parameter *K* to circumvent overshooting of the system.
(2) The influence of heading deviation on gain parameter *K* is obtained by trial-and-error method, whereby a larger heading deviation corresponds to a larger gain parameter *K*, and vice versa, and as illustrated in Figure 7, the fuzzy rule relationship of lateral error, heading error and *K*. In this paper, 49 fuzzy inference rules are summarised and a fuzzy inference rule table is formulated, as shown in Table 1.

## 4. Fuzzy PID Controller Design and Verification

### 4.1. Fuzzy PID Controller Design

In the preceding analysis, the control of the path tracking algorithm K is managed by the fuzzy algorithm. However, during the movement of the mining vehicle, the control of its longitudinal speed stability and the heading angle calculated by the algorithm may differ from that in the actual process. This section will research the longitudinal control of the deep-sea mining vehicle to ensure that the actual heading angle of the mining vehicle is the same as that obtained in the algorithm.

It is widely acknowledged that traditional PID controllers are distinguished by their simplicity, robustness, adaptability, and independence from model characteristics. However, conventional PID controllers are incapable of achieving optimal control performance for non-linear systems. It is evident that this section employs a fuzzy PID approach for the purpose of controlling the heading of the mining vehicle. The fuzzy PID controller has been shown to enhance control accuracy and response speed by incorporating a fuzzy module that dynamically adjusts the PID control coefficients based on error magnitude and error rate. This approach has been demonstrated to improve both control precision and responsiveness.

The heading angle deviation e, deviation change ec, and the outputs are the fuzzy linguistic variables E, EC, and $K_{p},K_{i},K_{d}$ are the fuzzy variables of $k_{p},k_{i},k_{d}$. The E and EC theories are set to be {−3, −2, −1, 0, 1, 2, 3} and $K_{p},K_{i},K_{d}$ to be {−5, −4, −3, −2, −1, 0, 1, 2, 3, 4, 5}. The GAIN module, both before and after the fuzzy controller, functions as a scaling factor that converts the output to the range of the thesis.

**Figure 8:**
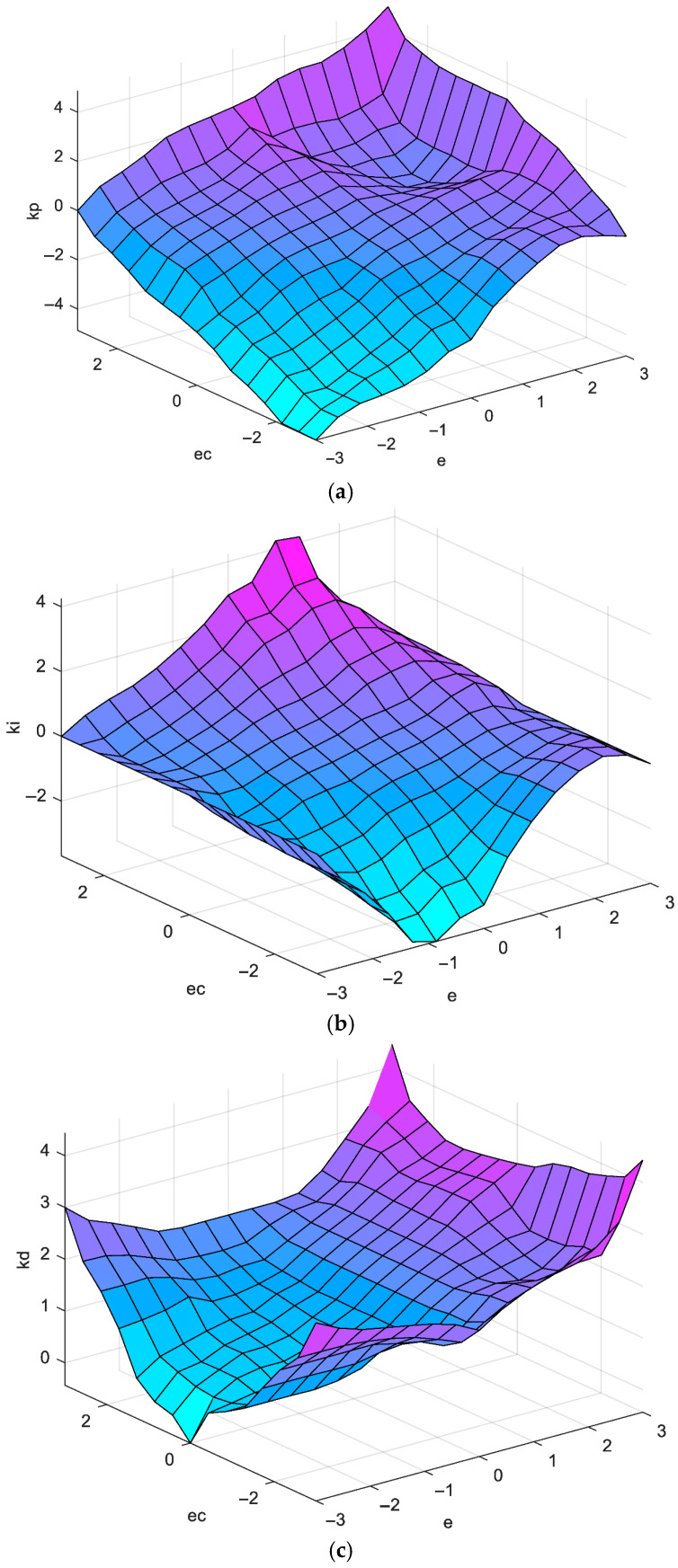
(**a**) Scale parameter *K_p_*; (**b**) scale parameter *K_i_*; (**c**) scale parameter *K_d_*.

The values of each linguistic variable of the E, EC and PID parameters are selected as follows: positive large for PB, positive medium for PM, positive small for PS, zero for ZO, negative small for NS, negative medium for NM, negative large for NB, and their respective fuzzy subset affiliation functions on the domain are triangular. The input-output relationship of the fuzzy controller is illustrated in Figure 8.

**Figure 9:**
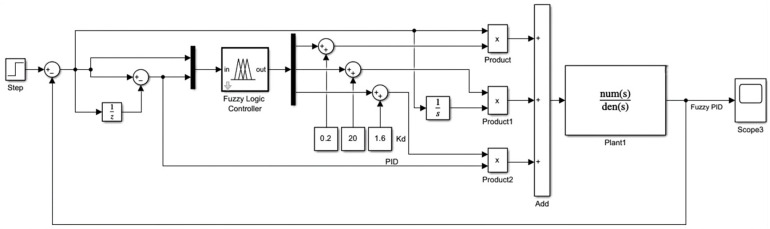
Fuzzy PID controller.

The implementation of fuzzy PID control is contingent upon the varying states of system operation, with consideration given to the interrelation of $K_{P}$, $K_{i}$ and $K_{d}$. Drawing upon engineering expertise, the fuzzy rectification of these three parameters is designed. The input linguistic variables are designated as error e and deviation rate of change ec. The values of linguistic variables are assigned {NB, NM, NS, ZO, PS, PM, PB} seven fuzzy values; select the output linguistic variables as $\Delta K_{p},\Delta K_{i},\Delta K_{d}$, the values of linguistic variables are also taken {NB, NM, NS, ZO, PS, PM, PB} seven fuzzy values, and establish the fuzzy rule table of $\Delta K_{p},\Delta K_{i},\Delta K_{d}$ as follows:

As illustrated in Figure 9, the fuzzy PID control block diagram comprises the following elements. Heading deviation e and its rate of change ec are designated as inputs to the control system. The fuzzy controller then dynamically adjusts the control parameter $K_{p},K_{i},K_{d}$ of the PID controller according to e and its rate of change ec. Consequently, the required angular difference is calculated through the PID controller.

The ultimate output parameter derived by the PID controller is the differential in rotational speed Δω between the two sides of the primary wheel.

(20) $$\Delta \omega =K_{p}\times e\times K_{i}\times \int edt+K_{d}\frac{de}{dt}$$

At this particular juncture, the target speed of the driving wheels of the mining truck is

(21) $$\left\{\begin{array}{l} \omega_{L}=\omega_{0}-\Delta \omega \\ \omega_{R}=\omega_{0}+\Delta \omega \end{array}\right.$$

**Figure 10:**
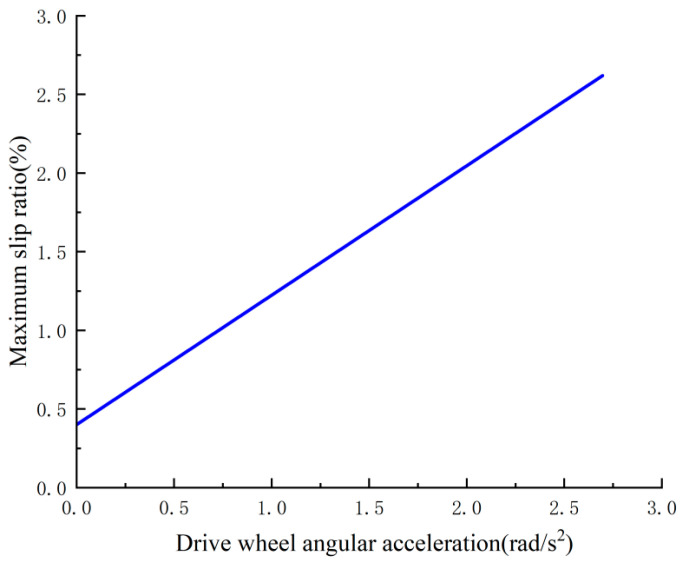
Relationship between angular acceleration of drive wheels and maximum slip rate.

As posited in the preceding study on the skidding rate, given the unfeasibility of directly measuring the angular acceleration of the driving wheel, it is imperative to focus on the skidding rate under various angular acceleration scenarios of the driving wheel. This approach enables the determination of the optimal angular acceleration for the mining vehicle, as illustrated in Figure 10, which delineates the correlation between the angular acceleration of the driving wheel and the maximum skidding rate.

### 4.2. Parameter Optimisation of Fuzzy PID Based on Adaptive Genetic Algorithm

Genetic algorithms have been demonstrated to optimise the fuzzy rules of fuzzy PID controllers, constituting a potent intelligent control strategy. The process of optimisation is achieved through the implementation of cyclical iterations of encoding, fitness evaluation, selection, crossover and mutation. These iterations enable the automatic and global search for optimal rule sets, thereby significantly enhancing the performance of the controller. The crossover operator is a simulation of the process of biological hybridisation, whereby parental genes are recombined in order to generate new offspring. The present paper employs genetic algorithms to optimise the proportional and quantisation factors requiring real-time adjustment in fuzzy control systems. The employment of these algorithms is founded upon the principles of fuzzy control theory, and the resulting parameters are shown to deliver superior output performance.

The specific process of genetic algorithms is described as follows:

(1) Chromosome Encoding In the field of genetic algorithms, prevalent encoding methodologies encompass binary encoding, natural number encoding, real number encoding, and tree-based encoding. The present paper proposes the utilisation of real number encoding as the coding scheme for the genetic algorithm. The quantisation factors and proportional factors requiring optimisation are treated as genes within individuals. The system perpetually performs optimisation on these parameters with a view to enhancing the performance of the original fuzzy controller.
(2) Secondly, the selection operation must be considered. The purpose of the selection operation is to select superior individuals during the evolutionary process. This operation determines which individuals will be passed on to the next generation and governs the diversity within the evolutionary process. In relation to the selection strategies employed, this paper utilises the tournament method. In each round, a random number of individuals are selected from the population. Subsequently, the most outstanding individuals are selected based on their performance, retained, and incorporated into the subsequent generation. This selection process is repeated cyclically until the size of the new population matches that of the original population. The tournament method’s key benefit is its capacity to effectively preserve superior individuals, minimise the influence of inferior ones, and promote population evolution through competitive selection.
(3) The following operations are to be conducted: crossover and mutation. The process of crossover entails the exchange of chromosomal segments between two parent individuals, thereby generating a new set of offspring. Mutation, on the other hand, involves the random alteration of an individual’s chromosomes, resulting in the introduction of novel genetic information.
(4) The termination condition utilises the number of evolutionary generations as the genetic algorithm’s termination parameter. Upon reaching the specified number of generations, the algorithm ceases operation and outputs the optimal individual within the current population as the optimal solution.

In this study, a total of 147 parameters require optimisation across Table 2, Table 3 and Table 4; all parameters are integers. Their value range is [1, 7], corresponding to [NB, NM, NS, Z, PS, PM, PB] in the optimisation process. Given the efficiency of MI-LXPM in integer-constrained optimisation problems, a real-coded genetic algorithm is employed to address this optimisation task. During motion, the objective function aims to minimise the expected path tracking error:

(22) $$\min f(e)=\sqrt{\frac{\sum_{i=1}^{n}{e_{i}}^{2}}{n}}$$

In genetic algorithms, the objective function is always the minimisation of the fitness function. Therefore, in the minimal objective function, the fitness function is expressed as:

(23) $$F\mathrm{it}(f(e))=\frac{1}{1+m-f(e)},m\geq 0,m-f(e)\geq 0$$

**Figure 11:**
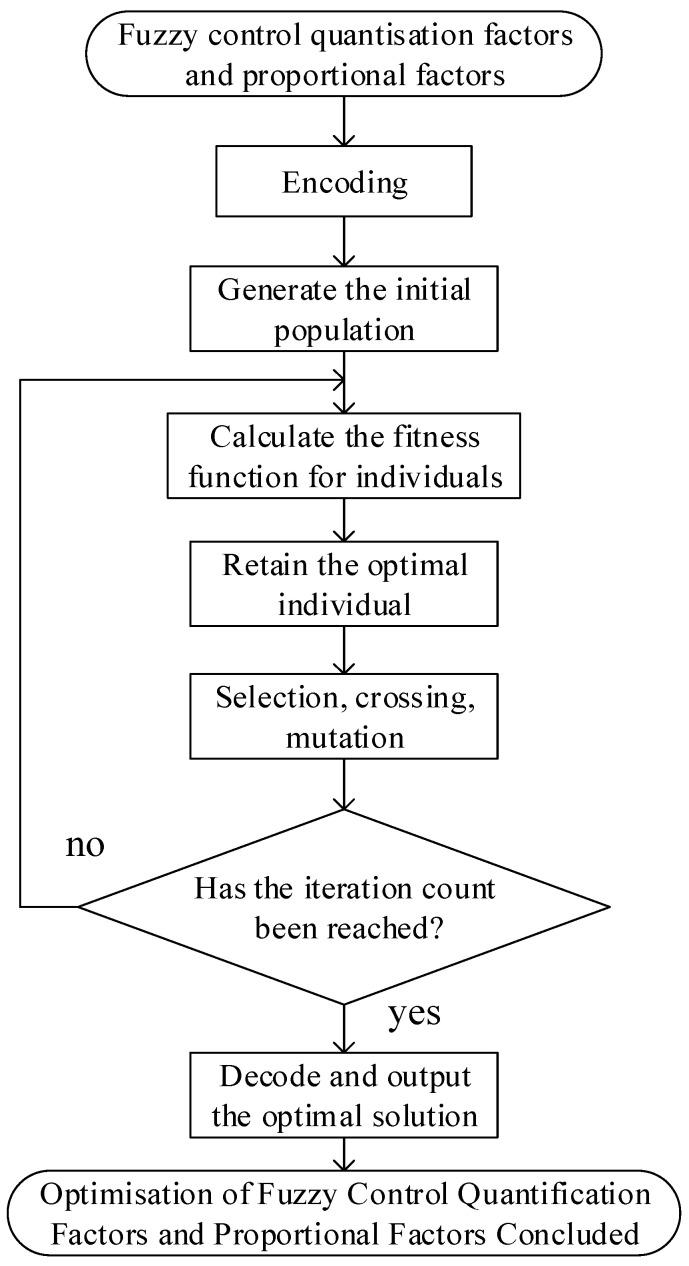
Fixed-Parameter Genetic Algorithm Flowchart.

Within the genetic optimisation algorithm framework, crossover and mutation operators function in synergy to facilitate both global and local searches of the search space. The construction of a fuzzy controller within the MATLAB/Simulink environment is presented, in conjunction with a joint simulation with a mining vehicle model. The fuzzy controller is optimised using a genetic algorithm. During the process of joint simulation, the genetic algorithm is responsible for updating parameters based on the value of the fitness function at each iteration. This process is repeated iteratively until convergence criteria are met and optimal parameter values are obtained. The optimisation workflow is illustrated in Figure 11.

Whilst standard GAs have been demonstrated to exhibit formidable global search capabilities in terms of parameter optimisation, it is evident that their performance remains to be significantly influenced by the selection of control parameters, such as the crossover probability $P_{c}$ and the mutation probability $P_{m}$. Fixed values for $P_{c}$ and $P_{m}$ have been shown to struggle to balance the algorithm’s exploration and exploitation capabilities: excessively high $P_{c}$ and $P_{m}$ may destroy promising solutions, causing the algorithm to degenerate into random search; conversely, excessively low values readily trap the population in local optima, leading to premature convergence. In order to surmount this limitation, the present paper puts forward a proposal for an adaptive genetic algorithm. This approach involves the dynamic adjustment of $P_{c}$ and $P_{m}$, contingent on the evolutionary state of the population. Consequently, this approach serves to both preserve diverse populations and safeguard high-quality individuals. The objective of this strategy is to achieve superior controller parameter solutions.

The fundamental principle of adaptive strategies is that superior individuals with fitness values above the population average should be subjected to reduced probabilities of crossover and mutation to safeguard their genetic structure from disruption. Conversely, individuals with sub-average fitness levels should be subject to a higher probability of genetic manipulation, with the objective of fostering the emergence of more advantageous genetic structures. It is asserted that, in accordance with the classical adaptive Formulae (24) and (25) proposed by M. Srinivas and L. M. Patnaik,

(24) $$P_{c}=\left\{\begin{array}{r} k_{1}\frac{f_{\max}-f^{\prime }}{f_{\max}-f_{avg}},f^{\prime }\geq f_{avg}\\ k_{2},f^{\prime }<f_{avg} \end{array}\right.$$

Here, $f_{\max}$ and $f_{avg}$ denote the maximum and average fitness of the current population, respectively; $f^{\prime }$ represents the parent with the higher fitness among the two parents to be crossed over; $k_{1}$ and $k_{2}$ are constants within the interval (0, 1], with $k_{1}=k_{2}=1$ adopted in this paper. This mechanism further preserves that parents with higher fitness functions exhibit lower crossover probabilities.

(25) $$P_{m}=\left\{\begin{array}{r} k_{3}\frac{f_{\max}-f}{f_{\max}-f_{avg}},f\geq f_{avg}\\ k_{4},f<f_{avg} \end{array}\right.$$

Here, f denotes the fitness of the individual undergoing mutation; $k_{3}$ and $k_{4}$ are constants within the interval (0, 1], with $k_{3}=k_{4}=0.5$ adopted in this paper. As before, this formula safeguards superior individuals while imposing greater mutational pressure upon inferior ones.

**Figure 12:**
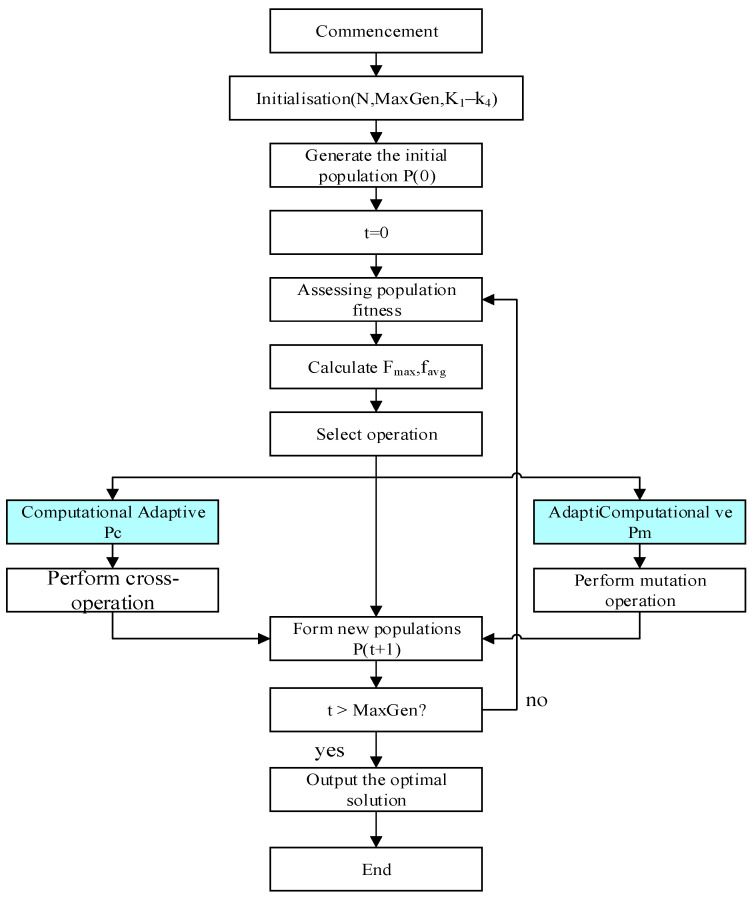
Adaptive GA Flowchart.

The adaptive genetic algorithm workflow is illustrated in Figure 12. In contradistinction to the standard parameter genetic algorithm, the primary distinction lies in the dynamic calculation of the fitness statistics $P_{c}$ and $P_{m}$ for each individual within the population prior to the execution of crossover and mutation operations. The primary steps of this algorithm are outlined below:

(1) Initialisation: Set the population size, maximum number of evolutionary generations (MaxGen), and randomly generate the initial population P(0).
(2) Fitness evaluation: Calculate the fitness value for each individual in the population, recording the maximum fitness $f_{\max}$ and average fitness $f_{avg}$ for the current generation.
(3) Selection: Employ tournament selection to choose parent individuals from the current population, forming a mating pool.
(4) Adaptive Crossover: For each pair of parents in the mating pool, calculate their crossover probability according to Equation (24) and execute the crossover operation.
(5) Adaptive Mutation: For each offspring generated by crossover, calculate its mutation probability using Equation (25) and execute the mutation operation.
(6) Form New Population: Combine the generated offspring to form the new generation population P(t + 1).
(7) Termination Check: If t<MaxGen, then set t=t+1 and return to step 2; otherwise, the algorithm terminates.

### 4.3. Chromosome Encoding Scheme for Adaptive Genetic Algorithms

The fuzzy PID controller designed here adjusts the proportional, integral and derivative gains in real time based on the tracking error e and its rate of change ec. The process of fuzzification is applied to both input variables, utilising a set of seven linguistic variables. It should be noted that the following abbreviations are employed: NB, NM, NS, ZE, PS, PM and PB. This process yielded 49 fuzzy rules, with each rule corresponding to one of the three output variables. These represent incremental adjustments to the fundamental PID gains.

In order to circumvent the utilisation of expert judgement or heuristic rule design, this paper treats all fuzzy rules—that is to say, all output values—as optimisation variables. In particular, the output of each rule is represented by a real number corresponding to the centroid position of the output membership function. Consequently, the total number of parameters to be optimised is:

(26) $$N=49\left(\Delta k_{p}\right)+49\left(\Delta k_{i}\right)+49\left(\Delta k_{d}\right)=147$$

The 147 parameters thus constitute the decision variables of the optimisation problem. The objective of the adaptive genetic algorithm is to search for the optimal parameter combination that minimises trajectory tracking error in a soft-bottom deep-sea environment.

The chromosome is encoded as a one-dimensional real-valued vector X, in the following form:

(27) $$X=\left[\underset{\Delta k_{p}}{\underbrace{x_{1},x_{2},\cdots ,x_{49}}},\underset{\Delta k_{i}}{\underbrace{x_{50},x_{51},\cdots ,x_{98}}},\underset{\Delta k_{d}}{\underbrace{x_{99},x_{100},\cdots ,x_{147}}}\right]$$

Each gene $x_{i}$ directly corresponds to the crisp output value of a specific fuzzy rule; this direct encoding strategy ensures a one-to-one correspondence between genotypes and controller behaviour, thereby eliminating the need for complex decoding transformations and significantly enhancing the algorithm’s transparency and reproducibility.

Subsequent to the convergence of the adaptive genetic algorithm, the optimal chromosome $X^{*}$ undergoes decoding during the offline phase in accordance with the following steps:

$$\begin{array}{l} X_{kp}=\left[x_{1},x_{2},\cdots ,x_{49}\right]\\ X_{ki}=\left[x_{50},x_{51},\cdots ,x_{98}\right]\\ X_{kd}=\left[x_{99},x_{100},\cdots x_{147}\right] \end{array}$$

Each segment is then entered into the corresponding fuzzy rule table (Table 2, Table 3 and Table 4) in line-priority order. During real-time control, the fuzzy inference engine employs these pre-optimised rule tables to compute $\left(\Delta K_{p},\Delta K_{i},\Delta K_{d}\right)$ using the centroid defuzzification method. These increments are then superimposed onto the nominal PID gain to form the final adaptive gain.

**Figure 13:**
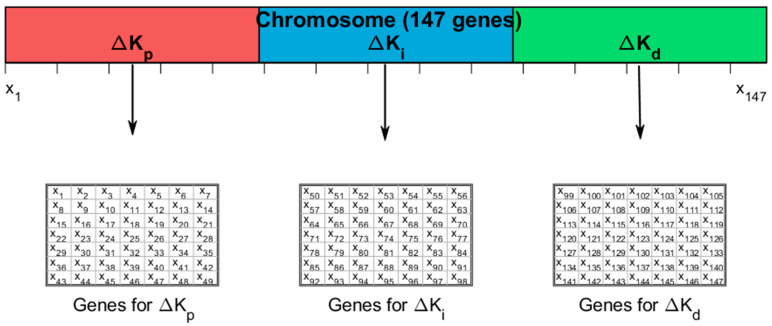
Chromosome structure for GA-based optimisation of fuzzy PID parameters.

As demonstrated in Figure 13, the upper segment of the image presents a horizontal bar chart that illustrates the complete chromosome, segmented into three portions, with each portion comprising 49 genes. The red segment corresponds to the $\Delta K_{p}$ genes $x_{1}$ to $x_{49}$, the blue segment to the $\Delta K_{i}$ genes $x_{50}$ to $x_{98}$, and the green segment to the $\Delta K_{d}$ genes $x_{99}$ to $x_{147}$. It is evident that these segments correspond to three independent fuzzy rule tables.

During system operation, construct a 101 × 101 two-dimensional lookup table (LUT) during the offline phase.

According to the parameters set in this paper, the population size P of the AGA population is set to 80.

### 4.4. Time Complexity Analysis

Prior to the initiation of simulations, it is imperative to undertake a time complexity analysis of both the Stanley path-tracking algorithm for the proposed technique and the fuzzy PID control optimised by the adaptive genetic algorithm. This will prevent simulation execution from becoming sluggish or exhibiting excessively long response cycles due to suboptimal choices.

The control framework proposed herein comprises two distinct logical phases: an offline optimisation phase and an online control phase, which exhibit markedly different temporal complexity characteristics. The control architecture incorporates two fuzzy inference systems, implemented via lookup tables: a fuzzy Stanley path-following controller and a fuzzy PID controller, which has been optimised by an adaptive genetic algorithm. The computational complexity analysis is as follows:

(1) Fuzzy Stanley Controller (Online):

The inputs to the fuzzy Stanley path-following algorithm are defined as follows: lateral deviation e and heading deviation $\theta_{e}$. Each input variable is divided into m=7 fuzzy sets. The total number of rules constituting the rule base is expressed as $m^{2}$, with a value of 49.

The fuzzy control is configured for offline processing, where a two-dimensional lookup table (LUT) is constructed. The construction of a 101 × 101 two-dimensional lookup table is achieved through the uniform sampling of K = 101 points across the input domain. This precision ensures that the error between the online interpolation results and the original fuzzy inference outputs remains within an acceptable range. The computational complexity of this process is $O\left(K^{2}\right)$, yet it is executed merely once.

During system operation, for any given input $\left(e,\theta_{e}\right)$, the controller queries the output value from the LUT via bilinear interpolation. The operation comprises two array indexing operations, four multiplications, and three additions for the interpolation calculation. The number of these operations is constant and independent of the number of fuzzy sets m. Consequently, its precise online time complexity is constant $O\left(1\right)$.

(2) Fuzzy PID Controller (Online):

The fuzzy PID controller takes as inputs the speed error (e) and the error rate of change (ec). Each input variable is divided into n=7 fuzzy sets. The rule base comprises a total of $n^{2}=49$ rules.

Construct a 101 × 101 two-dimensional lookup table (LUT) during the offline phase, with a complexity of $O\left(K^{2}\right)$.

During system operation, the system under discussion is analogous to the fuzzy Stanley controller, insofar as it employs bilinear interpolation to query the adjustment quantities for three outputs $\left(\Delta K_{p},\Delta K_{i},\Delta K_{d}\right)$ within the LUT. For each output, the interpolation operation is defined as $O\left(1\right)$. It is evident that, with three independent outputs, the total operations amount to three times that of a single output, yet remains constant. Consequently, its precise online time complexity is also $O\left(1\right)$.

(3) Adaptive Genetic Algorithm Optimisation of Fuzzy Rule Bases

According to the parameters set in this paper, the population size P of the AGA population is set to 80, the maximum generation G at 150, and the chromosome length L at 147. Each generation necessitated the exhaustive simulation of 80 individuals, with the simulation complexity of each individual being $O\left(S\right)$. Moreover, the calculation of the population mean fitness $f_{avg}$ and maximum fitness $f_{\max}$ necessitates the traversal of the entire population, with a complexity of $O\left(P\right)$. The calculation of each individual’s personalised $P_{c}$ and $P_{m}$ is contingent upon one or more arithmetic operations, which are based on the predefined fitness Formulae (24) and (25). It is imperative to note that, given the necessity to execute this operation on all P individuals, the complexity of the operation is $O\left(P\right)$.

Therefore, the total time complexity for the combination of all operations in the context of adaptive genetic algorithm optimisation can be expressed as follows:

(28) $$O\left(G\cdot \left(P\cdot S+P\cdot L+P\right)\right)=O\left(G\cdot P\cdot \left(S+L+1\right)\right)$$

Herein, S is primarily determined by the simulation duration and step size. In this paper, the step size is set to T = 0.01, while the duration is adjusted according to simulation requirements.

Since the constant term 1 can be absorbed in big O notation, it ultimately simplifies to

$$O\left(G\cdot P\cdot \left(S+L\right)\right)$$

In summary, the computational overhead of the adaptive genetic algorithm optimisation module is excessive, and it is therefore configured to run offline.

### 4.5. Experimental Verification and Analysis

The present experiment compares a fixed-parameter genetic algorithm with an adaptive genetic algorithm. First, the experimental group used an adaptive genetic algorithm, while the control group employed a fixed-parameter genetic algorithm. It is important to note that both algorithms shared identical evolutionary generations, initial population size, fitness function, and selection operator. This was performed to ensure rigorous comparative testing. The specific parameters of the genetic algorithm are listed in Table 5. The simulation duration may be adjusted according to the study’s requirements. Regarding the selection of P and G, a comprehensive review of existing literature indicates that settings of P = 50–100 and G = 100–200 are commonly used. Following this preliminary investigation, a series of experiments were conducted to explore the effects of varying combinations of P and G values. After a thorough analysis, it was determined that the combination of P = 80 and G = 150 produced stable and superior optimisation results within a reasonable computational time frame.

**Figure 14:**
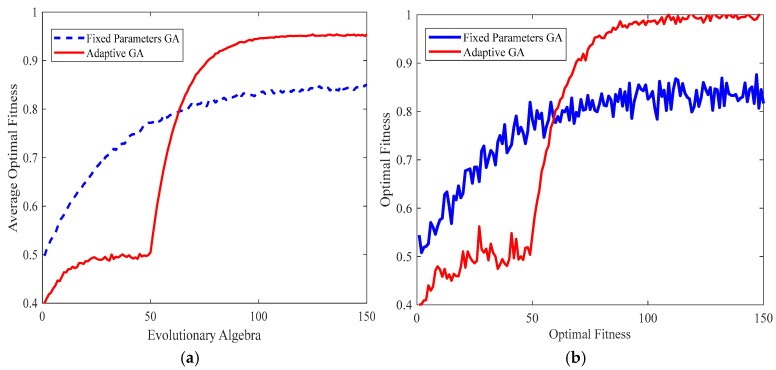
(**a**) Comparison of fitness values between the two algorithms; (**b**) Comparison of average optimal fitness values between the two algorithms.

As demonstrated in Figure 14a, the fitness values of the adaptive and fixed-parameter genetic algorithms are plotted against the increasing evolutionary generations. It has been demonstrated that, in comparison with the fixed-parameter GA, the adaptive GA displays considerably diminished fitness values during more abbreviated evolutionary periods. However, as the evolutionary generations increase, the fixed-parameter GA converges and stabilises around 0.825. By contrast, the adaptive GA converges at an evolutionary generation of 100, achieving an adaptability value of 0.95. As illustrated in Figure 14b, the mean optimal adaptability values of the two approaches increase with evolutionary generations. It is evident that the adaptive GA ultimately yields a significantly higher average optimal adaptability value than the fixed-parameter GA.

As demonstrated above, while the fixed-parameter GA exhibits faster convergence than the adaptive GA, it is susceptible to becoming trapped in local optima. Conversely, the adaptive GA, through a balanced approach to exploration and exploitation, circumvents the issue of premature convergence despite its protracted convergence process. The search ultimately converges to a higher-quality global optimum solution, thereby demonstrating superior global search capabilities.

### 4.6. Optimised Fuzzy Rules and Comparative Experiments

After 142 generations of evolution, the adaptive genetic algorithm obtained the global optimum solution. The optimised fuzzy rules are presented in Table 6, Table 7 and Table 8.

The aforementioned simulations have demonstrated the superiority of the adaptive genetic algorithm over the fixed-parameter genetic algorithm, and have yielded a fuzzy rule base optimised through the adaptive genetic algorithm. To validate the speed stability and heading control performance of the optimised fuzzy PID controller, a comparison is made here between the conventional fuzzy PID control algorithm and the optimised fuzzy PID controller.

**Figure 15:**
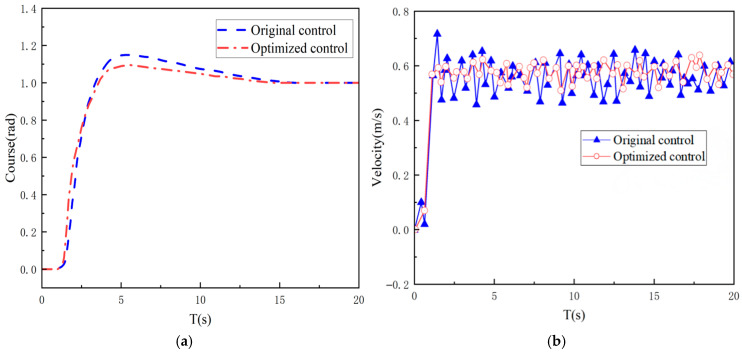
(**a**) Fuzzy PID with conventional PID stepwise correspondence; (**b**) Speed stability comparison.

The step signal is utilised as the input to facilitate a comparison and analysis of the respective situations of the two controllers for heading angle control, as illustrated in Figure 15. In comparison with the traditional PID controller, the fuzzy PID controller demonstrates superior performance in its response to the step signal, characterised by a reduced over-zero time and a diminished overshooting amount. The speed fluctuation is diminished in the presence of external interference, and the speed exhibits enhanced stability during the motion process. Consequently, the fuzzy PID controller exhibits enhanced anti-interference capabilities and superior robustness, rendering it particularly well-suited for the precise control of mining vehicle.

## 5. Simulation Analysis

As demonstrated in the preceding sections, this paper has effectively established a lateral and longitudinal path tracking control system that is well-suited for utilisation in deep-sea mining vehicles. Within this system, two controllers manage orthogonal control dimensions, respectively, forming a typical lateral-longitudinal control framework. The fuzzy Stanley path tracking algorithm is responsible for controlling lateral motion by determining the correct steering angle, while the fuzzy PID controller, which has been optimised via genetic algorithms, regulates the mining robot’s forward speed. The act of steering a vehicle is accomplished by means of adjusting the velocity differential between the left and right tracks. The target steering angle output by the fuzzy Stanley controller is converted into a track speed differential command. This differential is then superimposed with the total speed reference value output by the fuzzy PID controller, ultimately generating independent target speeds for each track.

**Figure 16:**
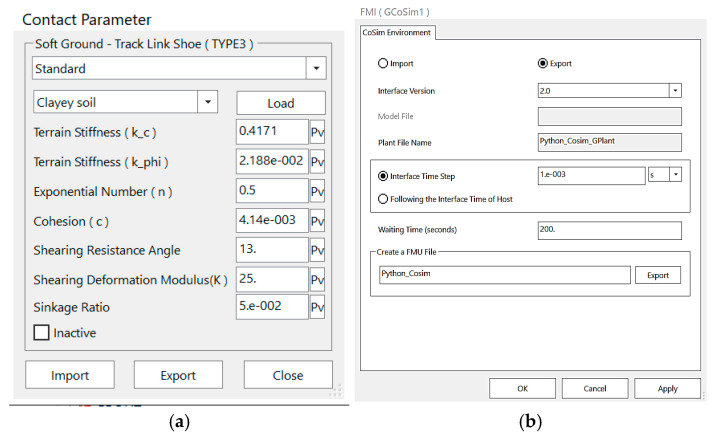
(**a**) Ground model parameters; (**b**) FMI file configuration.

**Figure 17:**
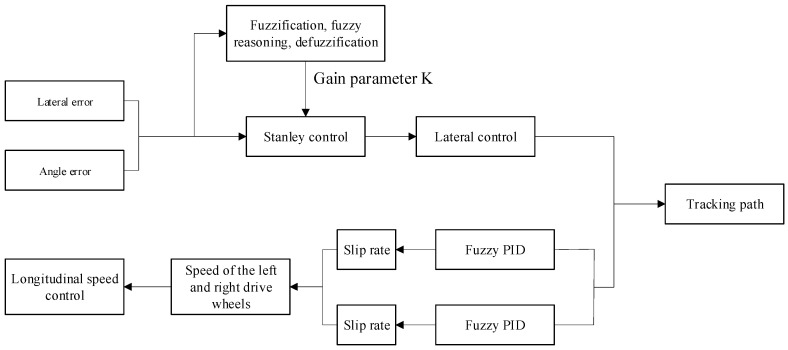
Simulation flowchart.

We employ the co-simulation between RecurDyn and Matlab/Simulink via the Functional Mock-up Interface (FMI) capability built into RecurDyn [27]. The simulation environment parameters are configured according to the parameters shown in Figure 16. Figure 16a presents the ground model parameters, while Figure 16b displays the configuration parameters of the FMI file. The parameter settings related to the mining vehicle and the seabed soil in the mining area are listed in Table 9. A schematic diagram of the simulation process is shown in Figure 17. The inputs of the FMI are defined as the rotational speeds of the left and right tracks, and the outputs include vehicle speed, heading angle, lateral coordinate, and longitudinal coordinate. The dynamic equations are derived in Matlab/Simulink, and numerical simulation analysis is conducted based on this model.

### 5.1. Straight-Line Travel Path

The overall simulation model is built in MATLAB/Simulink, defining the mining vehicle travelling along a straight path of Y = 10 m.

**Figure 18:**
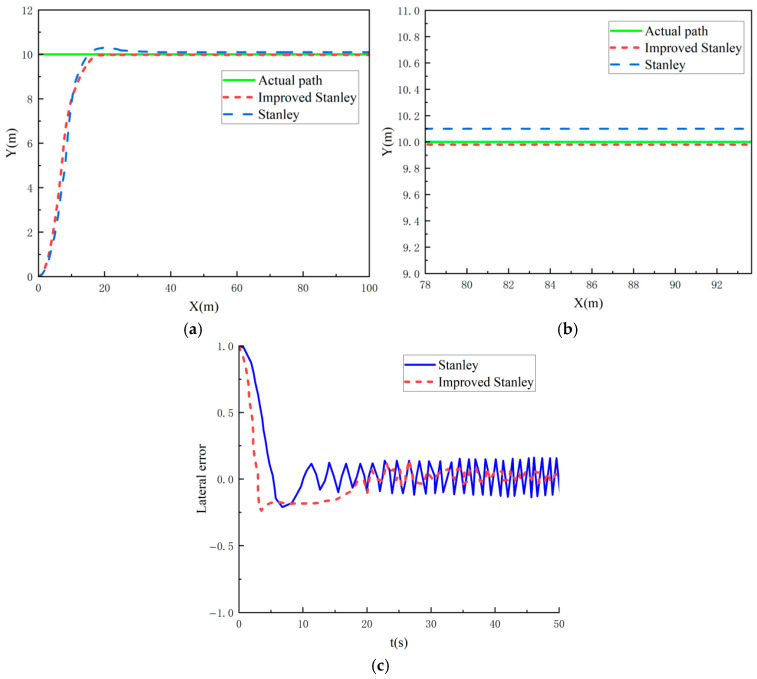
(**a**) Linear path simulation; (**b**) Local enlarged view of the linear path simulation; (**c**) Variation in error.

As shown in Figure 18, Figure 18a shows the comparison of the straight line travelling path tracking curves of Stanley’s algorithm and the improved Stanley’s algorithm, Figure 18b shows the local zoomed-in view of the straight line travelling path, and Figure 18c shows the tracking error curve. As demonstrated in Figure 18, both algorithms demonstrate effective tracking capabilities when simulating a straight-line path. However, when approaching the target path, the enhanced Stanley algorithm exhibits a maximum lateral error of approximately 0.2 m at 20 m of travel, with an average error of 0.1 m. Subsequent to this, the average error can be reduced to 0.01 m. m, while the Stanley algorithm has an increased lateral error at 20 m of travelling at the turning point, with a maximum lateral error of 0.2 m, an average lateral error of 0.1 m, and the error is in an oscillating state. Therefore, the improved Stanley algorithm can reach the target path more smoothly and accurately with less error oscillation.

### 5.2. Straight Line Lane Change

The trajectory of the mining vehicle is established within the MATLAB/Simulink module. The vehicle is initialised at the origin (0, 0), and its trajectory is delineated as follows:

(29) $$\left\{\begin{array}{cc} y=0 & 0\leq x\leq 10\\ y=x-10 & 10\leq x\leq 20\\ y=10 & x\geq 20 \end{array}\right.$$

The mining vehicle commences from the origin and executes two lane changes at 10 m and 20 m, respectively, with the two straight-line paths being 10 m apart.

**Figure 19:**
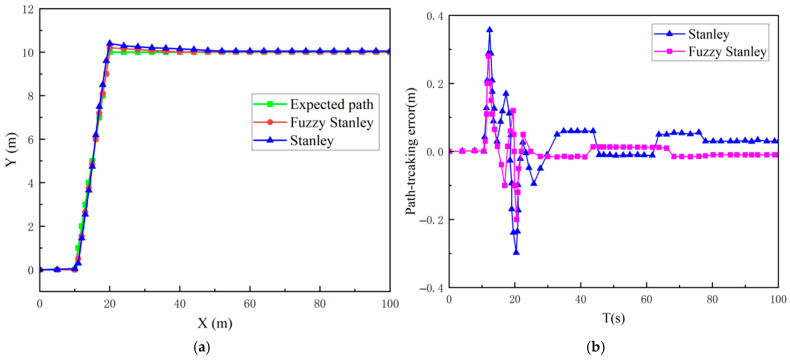

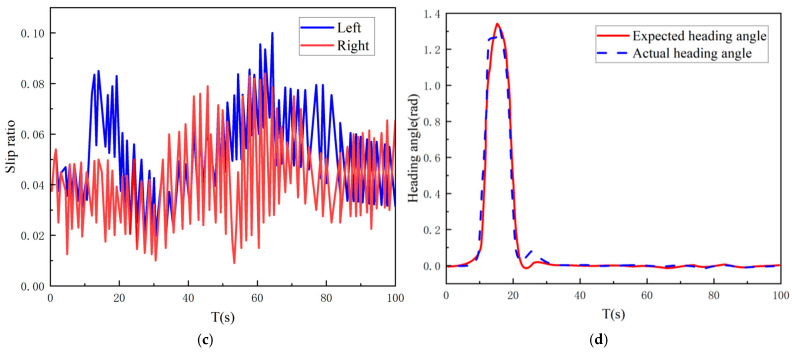
(**a**) Straight line lane change simulation; (**b**) error variation; (**c**) left track slip rate; (**d**) heading angle control effect.

As illustrated in Figure 19a, the straight-line lane-change trajectory of the mining vehicle is depicted, while Figure 19b provides a visualisation of the path-tracking deviation. Figure 19c presents the slip rate of the left and right tracks during the vehicle’s trajectory. As can be seen from Figure 18, the two controllers control well when performing straight-line path tracking, but after turning and changing lanes, it is obvious that the path tracking controller composed of the improved Stanley algorithm and fuzzy PID is more effective in straight-line lane-changing path tracking; At 10 m, the lateral error of the Stanley algorithm reached 0.38 m, whereas the improved Stanley algorithm exhibited a lateral error of 0.28 m. At 20 m, the lateral error of the Stanley algorithm was 0.25 m, while the improved Stanley algorithm recorded a lateral error of 0.2 m. After that, the average lateral deviation of the Stanley algorithm is 0.05 m, and the average lateral deviation of the improved Stanley algorithm is 0.01 m. It can thus be concluded that the Improved Stanley algorithm has a reduced lateral error and fluctuation range during lane changes (see Figure 19c). The figure shows the change in skidding rate of the left and right tracks of the mining vehicle during the exercise process. The maximum skidding rate of the left track is 9.8%, and the maximum skidding rate of the right track is 8.2%. It is evident that the tracked mining vehicle’s turning is predominantly influenced by the differential rotational speeds of the drive wheels on both sides. Consequently, the slipping rate of the tracks on both sides undergoes substantial changes during lane changes, with both slips remaining within the acceptable range, thereby effectively limiting slippage. As illustrated in Figure 19d, the path tracking controller in this paper effectively regulates the heading angle of the mining vehicle during lane changing manoeuvres.

### 5.3. Compound Curve Motion

The path is defined as:

(30) $$\left\{\begin{array}{ll} y=0 & 0\leq x\leq 20\\ {\left(x-20\right)}^{2}+{\left(y-20\right)}^{2}=400 & 10\leq x\leq 20\\ y=40 & x\geq 20 \end{array}\right.$$

A mining truck starts from the origin (0, 0) and travels 20 m in a straight line in circular motion, then continues in a straight line with a distance of 40 m between the two straight lines.

**Figure 20:**
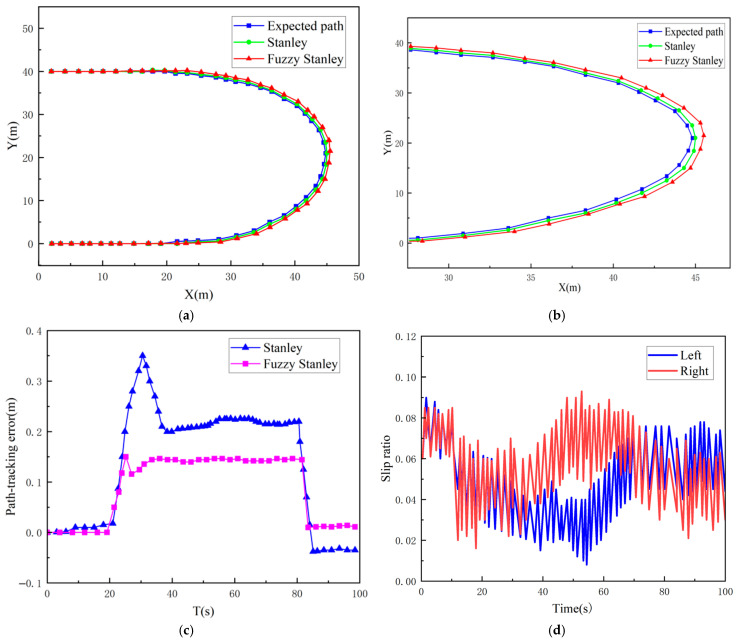
(**a**) Composite curve simulation; (**b**) local enlargement of the composite curve; (**c**) tracking error variation; (**d**) track slip rate on the left and right sides.

As illustrated in Figure 20a,b, the simulation results and local enlargement of the mining vehicle under the composite curved path are presented. Figure 20c demonstrates the tracking error of the mining vehicle path. As demonstrated in Figure 20, the discrepancy between the two controllers is negligible in linear movement, yet the mining vehicle is required to adjust its heading continuously during prolonged curved trajectory. The enhanced Stanley algorithm exhibits superior tracking capabilities. With respect to tracking error, the maximum deviation of the Stanley algorithm is 0.35 m, with a minimum deviation of 0.05 m, which stabilises after 80s, and the tracking error fluctuates above and below 0.05 m. The modified Stanley algorithm exhibits an enhanced tracking effect, with a tracking error of 0.05 m. 0.05 m fluctuation; the enhanced Stanley algorithm has a maximum error of 0.3 m, a minimum tracking error of 0.01 m, and the tracking error region is stable after 80s, and finally maintains at 0.01 m. As illustrated in Figure 20d, the slipping rate of the left and right tracks is shown, with the maximal slipping rate of 9% and 8.5% for the left and right tracks, respectively, which is in the range of the reasonable slipping rate.

### 5.4. Longitudinal Control Performance Comparison

To directly compare the heading control effects of the fuzzy PID optimised by the adaptive genetic algorithm (AGA) and the fixed-parameter genetic algorithm (FGA), two typical working conditions were configured to comprehensively evaluate the controller performance, namely the straight-line lane change condition mentioned above and the circular path condition with a radius of 5 m. The straight-line lane change condition is mainly used to verify the controller’s ability to maintain the set longitudinal velocity and track the target heading angle rapidly and stably during the dynamic process involving direction changes. The circular path tracking condition with a radius of 5 m is designed to validate the controller’s capacity to preserve velocity stability and heading angle accuracy under the stringent condition of continuous steering.

**Figure 21:**
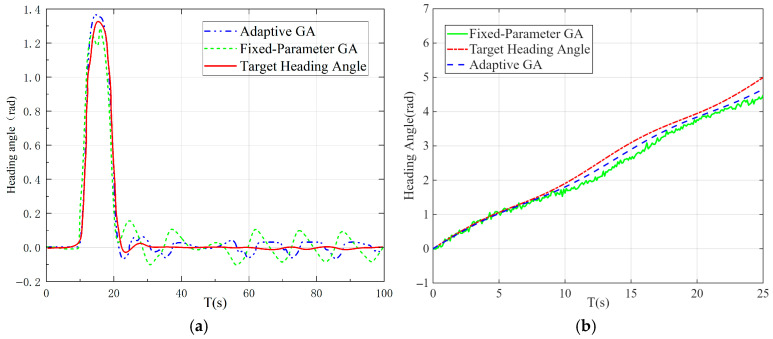
(**a**) Heading tracking under straight lane-changing conditions; (**b**) Heading tracking under circular path conditions.

As illustrated in Figure 21, Figure 21a depicts the heading tracking performance under the straight-line lane change condition, while Figure 21b shows that under the circular path condition. It can be observed from Figure 21a that two abrupt changes occur in the target heading angle during the lane change process, which are caused by the switching of target points. Such abrupt changes can be mitigated by adjusting the pre-steering point of the mining vehicle. Both algorithms exhibit a certain degree of lag in tracking the target heading but still maintain favourable control performance. Nevertheless, compared with the fuzzy PID controller optimised by the fixed-parameter genetic algorithm, the fuzzy PID controller optimised by the adaptive genetic algorithm demonstrates faster response speed and smaller overshoot in heading angle tracking. In Figure 21b, each jump in the target heading corresponds to a target point switch. Combined with the heading error analysis, it can be seen that during the tracking process of the two algorithms, the heading error of the fuzzy PID controller optimised by the fixed-parameter genetic algorithm is relatively large, reaching 0.06 rad. In contrast, the fuzzy PID controller optimised by the adaptive genetic algorithm can better coordinate the speed of the tracks on both sides, thereby achieving more precise heading angle tracking on the circular path with a significantly reduced tracking error.

The simulation results that have been referenced demonstrate the effectiveness of the proposed method. In order to assess its performance, the time required to run the programme on a given dataset was measured, with a view to ensuring that it operates with sufficient speed.

Firstly, the adaptive genetic algorithm employed to optimise the parameters of the fuzzy PID controller utilised the following settings: a population size of 80 and a maximum number of evolutionary generations of 150. The simulation concluded after 142 generations. Preliminary calculations indicate that the entire optimisation process requires approximately 48.6 min. Notwithstanding the fact that this process consumes a considerable amount of computational resources, it is solely offline optimisation which is concerned with, and thus does not impact the controller’s overall computational capability. It is noteworthy that all the simulation experiments referenced above employed a time step of 0.01 s. The execution times of the two core controllers were measured through testing. The execution time of the fuzzy Stanley path-following algorithm was found to be 0.42 milliseconds on average, while the fuzzy PID controller exhibited an average execution time of 0.38 milliseconds per step. Consequently, the total average time for the entire hybrid control strategy within a single control cycle amounted to a mere 0.8 milliseconds. This reduction in the control cycle time is significant, as it demonstrates the system’s exceptional efficiency in accomplishing tasks in real time.

## 6. Conclusions

This paper, based on a review of extensive domestic and international literature concerning path tracking algorithms for deep-sea mining vehicles, undertakes a systematic investigation into path tracking issues for such vehicles. The path tracking problem is composed of three constituent elements: analysis of the deep-sea mining vehicle model, path tracking algorithms, and heading controllers. Research is conducted separately in each of these three areas. The specific content is as follows:

(1) This paper employs a two-degree-of-freedom mining vehicle kinematic model and improves the traditional Stanley algorithm based on this model. The fuzzy algorithm is utilised to control the size of the gain parameter K in the Stanley algorithm, thereby enhancing the mining vehicle’s capacity to adapt to complex paths.
(2) The establishment of a fuzzy PID controller was achieved, with its fuzzy parameters being optimised using an adaptive genetic algorithm. This approach was adopted to address the drawbacks of premature and slow convergence, which are inherent in standard genetic algorithms. The experimental results demonstrate that the controller optimised by this method exhibits superior speed, stability and enhanced robustness compared to conventional approaches.
(3) A motion simulation model for the mining robot was established using MATLAB/Simulink software, based on the dynamic model of the deep-sea mining robot and the properties of seabed soft mud. A simulation analysis was conducted on several typical path-following problems. The simulation results demonstrate that, in comparison with the traditional Stanley algorithm and fuzzy PID control, this method exhibits superior path-tracking capability.

Despite the path tracking controller demonstrating effective tracking capabilities, there are still a number of issues that require resolution.

(1) The accuracy of the mining vehicle dynamic model is compromised due to the constraints imposed by soil research. In real-world mining operations, the soil characteristics encountered by the mining vehicle are not static. The design of the control algorithms for the selection This may not always yield satisfactory results for different soil properties. Regarding the solution, the employment of recursive least squares or an extended Kalman filter (EKF) may facilitate the real-time estimation of the current soil shear strength or internal friction angle through the observation of the vehicle’s slip rate, drive torque, and speed. The real-time soil parameters obtained from the estimator are fed directly into the fuzzy PID controller.
(2) The fuzzy PID heading controller utilises only the difference between the two sides of the track as the control quantity input. In practice, it takes time for the mining vehicle to reach the difference between the two sides of the tracks, and for the control input speed to reach the desired value from the current speed of the tracks. This process also requires a control algorithm.
(3) The present study is lacking in validation through actual experimentation. While simulation models can demonstrate algorithmic stability to a certain extent, discrepancies between simulation and reality persist. Notwithstanding, this approach does provide a sound solution. Future hardware-in-the-loop testing may be conducted to validate the real-time performance of algorithms on actual hardware. Ultimately, full-system testing will be performed in controlled experimental environments such as large-scale water tanks.
(4) The present study compares the proposed algorithm solely with the conventional Stanley method and traditional fuzzy PID control. While significant improvements over these approaches are demonstrated, a comparison with advanced control strategies that are widely employed in subsea vehicle research is absent. Subsequent investigations will concentrate on thorough performance comparisons with state-of-the-art control strategies, such as model predictive control and sliding mode control. This will facilitate a more precise evaluation of the method’s advantages and limitations in complex underwater path-following tasks.

## Figures and Tables

**Table 1:** Table rule inference table.

$e\backslash \theta_{e}$	NB	NM	NS	ZO	PS	PM	PB
NB	M	CS	S	VS	S	CS	M
NM	CD	M	CS	S	CS	M	CD
NS	B	CD	M	CS	M	CD	B
ZO	VB	B	CD	M	CD	B	VB
PS	B	CD	M	CD	M	CD	B
PM	CD	M	CS	S	CS	M	CD
PB	M	CS	S	VS	S	CS	M

**Table 2:** Scale parameter $K_{p}$ fuzzy rule table.

e\ec	NB	NM	NS	ZO	PS	PM	PB
NB	NB	NB	NM	NM	NS	NS	ZO
NM	NB	NM	NM	NS	NS	ZO	PS
NS	NM	NM	NS	NS	ZO	PS	PS
ZO	NM	NS	NS	ZO	PS	PS	PM
PS	NS	NS	ZO	PS	PS	PM	PM
PM	NS	ZO	PS	PS	PM	PM	PB
PB	ZO	PS	PS	PM	PM	PM	PB

**Table 3:** Scale parameter $K_{i}$ fuzzy rule table.

e\ec	NB	NM	NS	ZO	PS	PM	PB
NB	NB	NB	NM	NM	NS	ZO	ZO
NM	NB	NB	NM	NS	NS	ZO	ZO
NS	NB	NM	NS	NS	ZO	PS	PS
ZO	NM	NM	NS	ZO	PS	PM	PM
PS	NM	NS	ZO	PS	PS	PM	PB
PM	ZO	ZO	PS	PS	PM	PB	PB
PB	ZO	ZO	PS	PM	PM	PB	PB

**Table 4:** Scale parameter $K_{d}$ fuzzy rule table.

e\ec	NB	NM	NS	ZO	PS	PM	PB
NB	PS	NS	NB	NB	NB	NM	PS
NM	PS	NS	NB	NM	NM	NS	ZO
NS	ZO	NS	NM	NM	NS	NS	ZO
ZO	ZO	NS	NS	NS	NS	NS	ZO
PS	ZO	ZO	ZO	ZO	ZO	ZO	ZO
PM	PB	NS	PS	PS	PS	PS	PB
PB	PB	PM	PM	PM	PS	PS	PB

**Table 5:** Genetic Algorithm Simulation Parameters.

Parameter Category	Parameter Values
Code Length	147
Population Size	80
Number of Generations	150
Evolution Probability	0.8
Mutation Probability	0.01
Crossover Probability Range	[0.6, 0.9]
Mutation Probability Range	[0.01, 0.1]
Simulation Duration	100
Simulation Step Size	0.01

**Table 6:** Optimised proportional parameter $K_{p}$ fuzzy rule table.

e\ec	NB	NM	NS	ZO	PS	PM	PB
NB	NB	NB	NM	NM	NS	NS	ZO
NM	NB	NM	NM	NS	NS	ZO	PS
NS	NM	NM	NS	NS	ZO	PS	PS
ZO	NM	NS	NS	ZO	PS	PS	PM
PS	NS	NS	ZO	PS	PS	PM	PM
PM	NS	ZO	PS	PS	PM	PM	PB
PB	ZO	PS	PS	PM	PM	PM	PB

**Table 7:** Optimised proportional parameter $K_{i}$ fuzzy rule table.

e\ec	NB	NM	NS	ZO	PS	PM	PB
NB	NB	NM	NS	ZO	NS	ZO	NS
NM	NM	ZO	ZO	PS	NS	PS	ZO
NS	NS	ZO	ZO	PM	ZO	PS	ZO
ZO	ZO	PS	PM	PB	PS	PS	ZO
PS	ZO	PS	PM	PM	PS	ZO	NS
PM	ZO	PS	PS	PS	PM	ZO	NM
PB	NS	ZO	ZO	ZO	PM	NM	NB

**Table 8:** Optimised proportional parameter $K_{d}$ fuzzy rule table.

e\ec	NB	NM	NS	ZO	PS	PM	PB
NB	NB	NB	NM	NS	NS	NS	ZO
NM	NB	NM	NS	NS	NS	NS	ZO
NS	NM	NS	NS	ZO	ZO	ZO	ZO
ZO	NM	NM	NS	ZO	PS	PM	PM
PS	ZO	ZO	ZO	ZO	PS	PS	PM
PM	ZO	PS	PS	PS	PS	PM	PB
PB	ZO	PS	PS	PS	PM	PB	PB

**Table 9:** Mining vehicle parameters and marine mine and soil parameters.

Parameter Name	Notation	Parameter Value
Mining Vehicle Quality	M	6000 kg
Track centre distance	D	1.9 m
Track grounding length	L	2.66 m
Track width	B	0.6 m
Track grounding area	A	1.596 m^2^
Water resistance area	$A_{2}$	2.2 m^2^
Moment of inertia of a vehicle body	I	8.24 kg∙m^2^
Soil cohesion in mining areas	C	5.4 kpa
Angle of internal soil friction	Φ	6.2°
Soil modulus of rigidity	K	15 cm
Positive soil friction coefficient	$\mu_{r}$	0.2
Soil lateral friction coefficient	$\mu_{l}$	0.55
Soil specific gravity	$\gamma_{S}$	12.2 KN/m^3^
Density of sea water	ρ	1070 g/m^3^
Coefficient of water resistance	$K_{s}$	0.009 kN∙m/(kg∙s)

## Data Availability

The original contributions presented in this study are included in the article. Further inquiries can be directed to the corresponding author.
